# *In vivo* gene therapy: A strategy for mutations, degenerations, and tumors

**DOI:** 10.1016/j.gendis.2025.101808

**Published:** 2025-08-19

**Authors:** Tao Wang, Mingyang Yu, Ping Liu, Zhiqiang Song, Cheng Li, Jianmin Yang, Na Liu

**Affiliations:** a Department of Hematology, Institute of Hematology, Changhai Hospital, Naval Medical University, Shanghai 200433, China; b Department of Joint Bone Disease Surgery, Changhai Hospital, Naval Medical University, Shanghai 200433, China; c Department of Orthopaedics, The 922nd Hospital of the Joint Service Support Force of the PLA, Hengyang, Hunan 421002, China

**Keywords:** Cancers, Degeneration-related diseases, Delivery vectors, DNA nucleases, Genetic mutation disorders, *In vivo* gene therapy

## Abstract

Gene mutations, organ function degeneration, and carcinogenesis are the primary threats to human health. Gene therapy, which involves the addition, deletion, regulation, and editing of genes, as well as the development of genetic vaccines, can potentially cure genetic mutation disorders, degenerative diseases, and cancers. *Ex vivo* gene therapy has recently been used to treat monogenetic mutation diseases of the hematopoietic system and cancers. However, *in vivo* gene therapy remains inapplicable. The primary elements of *in vivo* gene therapy include deoxyribonucleic acid (DNA) nucleases (*e.g.*, zinc finger nucleases, transcription activator-like effector nucleases), CRISPR-Cas system, base editors, prime editors, and delivery vectors (*e.g.*, viral and non-viral vehicles). According to the development of DNA nucleases and delivery vectors, *in vivo* gene therapy can be made available for future clinical use. The current review summarizes the development of DNA nucleases and delivery vectors for *in vivo* gene therapy, emphasizing recent progress.

## Graphical abstract

**Figure:**
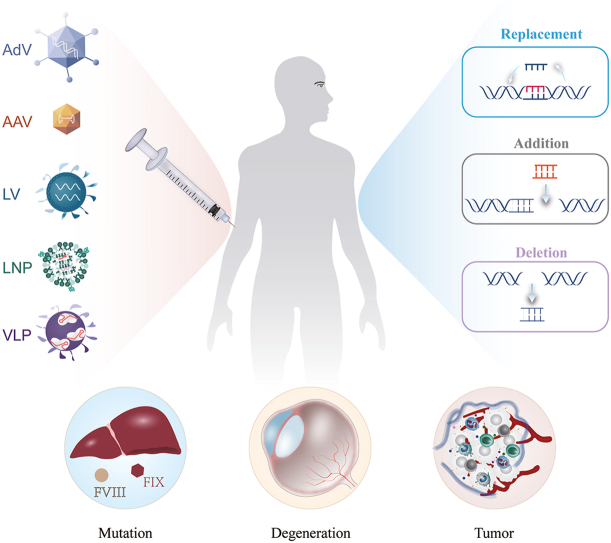

## Introduction

In 1972, Theodore Friedmann and Richard Roblin proposed that gene therapy may be a treatment option for some genetic diseases.^1^ Gene therapy is a biological treatment conducted by introducing exogenous therapeutic genes into target cells to correct genetic disorders or compensate for gene defects and abnormalities, and can be classified into four types: i) gene addition or deletion; ii) gene expression control; iii) gene editing or replacement; iv) deoxyribonucleic acid (DNA) or ribonucleic acid (RNA) vaccine.^2^ Ideally, gene therapy can durably express therapeutic genes efficiently and minimize undesired adverse events.^3^ Developing genome editing tools with high efficiency, convenience, and low toxicity has been a life goal that scientists are striving to achieve. In the 1990s, the landmark cure of adenosine deaminase-deficient severe combined immunodeficiency by transferring the *ADA* gene into T cells and hematopoietic stem and progenitor cells (HSPCs) via gene therapy in clinics led to an upsurge in gene therapy.^4^ With the development of next-generation sequencing, the responsible genes for 50% of >7000 estimated monogenetic diseases have been identified, which is a prerequisite for gene therapy.^5^ Besides these Mendelian disorders, some idiopathic diseases, such as age-related macular degeneration or tumors with specific antigens, can also be cured by gene delivery into target cells.^6^ There are two ways of gene delivery: *ex vivo* and *in vivo*. *Ex vivo* genome editing, *e.g.*, gene modification of HSPCs for severe combined immunodeficiency and gene editing of T cells, natural killer cells, or other immune cells for expressing specific antigen receptors for targeted tumor therapy, is the most widely adopted method in gene therapy.^7^^,^^8^ When manufacturing gene-modified cells *ex vivo*, cells must be collected, cultured, and activated for gene transduction. This process is time-consuming and expensive. Besides, genome editing *ex vivo* is inapplicable to diseases involving solid organs, including the eye and brain. Thus, gene editing *in vivo* shows great potency to cure these diseases. *In vivo* gene therapy has potential in regenerative medicine, including the nerve, myocardium, bone, and hair.^9^

Genome editing tools involve two main parts: a DNA nuclease and a gene delivery vector. DNA nucleases perform gene modification on specific sites by creating double-strand DNA breaks (DSBs) and repairing the DNA cut via non-homologous end joining (NHEJ) or homologous recombination DNA repair (HDR). NHEJ can occasionally insert or delete one or two bases, leading to a frameshift in the coding sequence to disrupt gene expression. This is often applied in loss-of-function mutations. HDR is realized by providing homologous arms on both sides of the target mutation site and DNA correction or modification based on the donor DNA templates.^10^ The development of base editors (BEs) and prime editors (PEs) enabled genome editing without DSB, which significantly increased the efficiency of clustered regularly interspaced short palindromic repeats associated with protein 9 (CRISPR-Cas9) and minimized the by-products of gene editing.^11^ To some extent, gene delivery vectors, including viral and non-viral vectors, determine the efficiency of genome editing and offer complementary advantages. Adeno-associated viruses (AAVs) and lentiviruses (LVs) are the most studied viral vectors. Considerable effort has been put into using AAVs for genome editing *in vivo*. AAV2 and AAV9 are approved by the US FDA for Leber congenital amaurosis type 2^12^ and spinal muscular atrophy.^13^^,^^14^ Their delivery *in vivo* has been proven safe and effective, making them promising prospects in gene therapy. However, in recent years, non-viral vectors, such as dextran-spermine polycation,^15^ lipid nanoparticles (LNPs),^16^ and virus-like particles (VLPs),^17^ have played an increasingly crucial role in nucleic acid and protein delivery. Besides, some researchers have utilized hydrophilic polymer nanoparticles to deliver proteins and small interfering RNA (siRNA), possessing a specific controlled-release system.^18^ Gene-silencing technology has been used to develop many other biodegradable polymers for siRNA delivery.^19^

This review provides an overview of the most recent advances in gene delivery *in vivo* and discusses their development, application, challenges, and future progress. We review the current application and highlight the outlook and challenges for gene therapy *in vivo*.

## Products and clinical trials of *in vivo* gene therapy

In 2003, the first commercial gene therapy product, Gendicine, was approved by the China FDA to treat head and neck squamous cell carcinoma. Gendicine is a recombinant human adenovirus carrying the p53 gene (by wild-type P53 gene delivery) to compensate for the mutated p53 function in cancer cells, suppressing tumor growth.^20^ Gene therapy is emerging as a promising therapeutic class for malignancies. Besides, oncolytic viruses are classical therapy models exploiting *in vivo* gene editing for malignancies. Modified viruses deliver multiple eukaryotic transgenes to tumor cells and express proteins to accelerate tumor cell death or induce anti-tumor immune response.^21^ Four oncolytic or non-oncolytic virus products have been approved for malignancy treatment. H101, an E1B gene-deletion adenovirus, is the first oncolytic virus product combined with chemotherapy that could significantly improve patient response to chemotherapeutic agents. It was approved by the China FDA in 2005 for nasopharyngeal carcinoma treatment.^22^^,^^23^ Talimogene laherparepvec (T-VEC), an engineered drug for herpes simplex virus type 1 (HSV1), selectively replicates in tumor cells and produces granulocyte-macrophage colony-stimulating factors inducing anti-tumor immune responses. It was approved by the US FDA in 2015 for recurrent melanoma treatment.^24^ Nadofaragene firadenovec or rAd-IFNa/Syn3, a recombinant adenovirus that carries human interferon alfa-2b complementary DNA (cDNA) for delivery into the bladder epithelium, was recently approved by the US FDA for bladder cancer treatment.^25^ Nadofaragene firadenovec has been assessed in a phase III clinical trial involving 198 patients with Bacillus Calmette-Guérin (BCG)-unresponsive, non-muscle-invasive bladder cancer. In this study, 157 patients received at least one dose of nadofaragene firadenovec. The complete response rate was 59.6% at 3 months, with a median duration of complete response of 7.31 months.^25^

**Figure 1:**
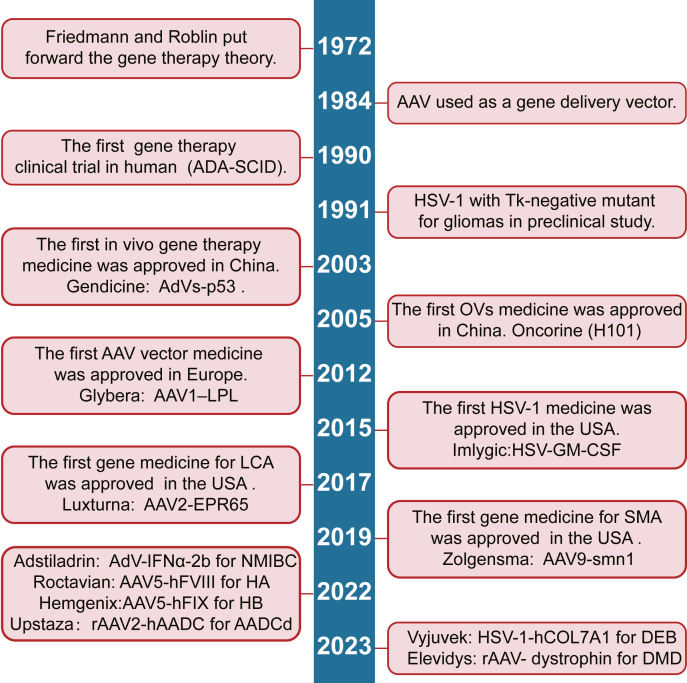
A timeline of *in vivo* gene therapy. Progress of *in vivo* gene therapy for use in malignancies, monogenic disorders, and degeneration disease. AAV, adeno-associated virus; TK, thymidine kinase; ADA-SCID, adenosine deaminase-deficient severe combined immunodeficiency; AdV, adenovirus; OV, oncolytic virus; HSV, herpes simplex virus; NMIBC, non-muscle invasive bladder cancer; LCA, Leber’s congenital amaurosis; HA, hemophilia A; HB, hemophilia B; AADCd, aromatic l-amino acid decarboxylase deficiency; DEB, dystrophic epidermolysis bullosa; DMD, Duchenne muscular dystrophy.

Recently, the United Kingdom Medicines and Healthcare products Regulatory Agency (MHRA) and US FDA approved exagamglogene autotemcel (exa-cel, Casgevy), a gene therapy product based on CRISPR-Cas9, to treat transfusion-dependent β-thalassemia and sickle cell disease.^26^ This gene therapy was conducted *ex vivo* by introducing CRISPR-Cas9 and guide RNA (gRNA) targeting B-cell lymphoma/leukemia 11A (*BCL11A*) to HSPCs through electroporation.^27^ However, for most monogenetic mutation diseases involving solid organs, *ex vivo* gene therapy is inapplicable, requiring *in vivo* gene therapy. The first clinical trial for *in vivo* gene editing utilizing CRISPR-Cas9 was performed with NTLA-2001.^28^ The trial was conducted to treat transthyretin amyloidosis. This disorder is a monogenetic mutation disease characterized by misfolded transthyretin (TTR) protein accumulation within tissues. NTLA-2001 is a gene therapy product with LNPs packing CRISPR-Cas9 messenger RNA (mRNA) and gRNA. It targets TTR and is delivered *in vivo* to knockout the TTR gene and ameliorate transthyretin amyloidosis. In the phase I clinical trial, including six transthyretin amyloidosis patients treated with NTLA-2001, the serum TTR protein concentration reduced by 52% on average in the group treated with 0.1 mg per kilogram and by 87% on average in the group treated with 0.3 mg per kilogram on day 28. These studies revealed that *in vivo* gene editing could potentially cure monogenetic mutation diseases with only mild adverse events. Figure 1 summarizes the development of *in vivo* gene therapy.

Besides monogenetic mutation disorders, *in vivo* gene therapy demonstrates potency in treating degeneration-related diseases, such as diseases of the eyes and brain. Table 1 summarizes the ongoing clinical trials of *in vivo* gene therapy.

**Table 1:** Ongoing clinical trials of *in vivo* gene therapy.

Vectors	Targets	Diseases	Gene therapy	Clinical trials
Adeno-associated virus	Eye	X-linked retinitis pigmentosa	*RPGR* gene	NCT03316560(Ⅰ)
		Non-syndromic retinitis pigmentosa	ChrimsonR-tdTomato gene	NCT03326336(Ⅰ)
		Retinitis pigmentosa	hPDE6B	NCT03328130(Ⅰ/Ⅱ)
		Retinitis pigmentosa	*RdCVF* and *RdCVFL*	NCT05748873(Ⅰ/Ⅱ)
		Neovascular age-related macular degeneration	Anti-VEGF protein	NCT05197270(Ⅰ)
				NCT05536973(Ⅲ)
				NCT05657301(Ⅰ/Ⅱ)
				NCT05672121(Ⅰ)
				NCT05984927(Ⅰ/Ⅱ)
		X-linked retinoschisis	*RS1* gene	NCT06066008(Ⅰ/Ⅱ)
		Diabetic retinopathy	*VEGF*-miRNA	NCT05930561(Ⅰ/Ⅱ)
	Liver	Hereditary angioedema	*hC1-INH*	NCT05121376(Ⅰ/Ⅱ)
		Wilson disease	*ATP7B* gene	NCT04884815(Ⅰ)
				NCT04537377(Ⅰ)
		Ornithine transcarbamylase deficiency	*OTC*	NCT05092685(Ⅰ/Ⅱ)
				NCT05345171(Ⅰ)
		Hemophilia A	FVIII	NCT06111638(Ⅰ/Ⅱ)
		Hemophilia B	FIX	NCT05152732(Ⅱ),
				NCT05709288(Ⅰ/Ⅱ)
		Hemophilia A/B	FVIII/IX	NCT05568719(Ⅰ/Ⅱ)
		Gaucher disease	Glucocerebrosidase	NCT05324943(Ⅰ)
	Neuron	Huntington’s disease	Huntington gene	NCT04120493(Ⅰ/Ⅱ)
				NCT05243017(Ⅰ)
		Tay-Sachs disease	β-hexosaminidase A or B cDNA	NCT04669535(Ⅰ)
		Multiple system atrophy	*GDNF*	NCT04680065(Ⅰ/Ⅱ)
		Canavan disease	Aspartoacylase	NCT04998396(Ⅰ)
		Alzheimer’s disease	Brain-derived neurotrophic factor	NCT05040217(Ⅰ/Ⅱ)
		Spasticity	Human AP4M1	NCT05518188(Ⅰ/Ⅱ)
		Parkinson disease	Glutamic acid decarboxylase	NCT05603312(Ⅰ/Ⅱ)
		Frontotemporal dementia	Granulin	NCT06064890(Ⅰ/Ⅱ)
		Friedreich ataxia	Frataxin	NCT05302271(Ⅰ)
		Duchenne muscular dystrophy	Dystrophin	NCT06114056(Ⅰ/Ⅱ)
	Heart	PKP2-related arrhythmogenic cardiomyopathy	Plakophilin-2a	NCT05885412(Ⅰ/Ⅱ)
		Hypertrophic cardiomyopathy	Myosin-binding protein C	NCT05836259(Ⅰ)
	Lung	Cystic fibrosis	Delete *CFTR*	NCT05248230(Ⅰ/Ⅱ)
	Ear	Congenital hearing loss	*OTOF*	NCT05788536(Ⅲ)
	Adrenal gland	Congenital adrenal hyperplasia	*CYP21A2* gene	NCT04783181(Ⅰ/Ⅱ)
Adenovirus	Tumor	Prostatic neoplasms	HSV-TK	NCT01913106(Ⅰ/Ⅱ)
		Glioblastoma	HSV-TK	NCT03603405(Ⅰ/Ⅱ)
		Solid tumor	TNF-α and IL-2	NCT04695327(Ⅰ)
		Melanoma	TNF-α and IL-2	NCT04217473(Ⅰ),
				NCT05222932(Ⅰ)
		Ovarian carcinoma	TNF-α and IL-2	NCT05271318(Ⅰ)
		Pancreas cancer	yCD/mutTKSR39rep-ADP	NCT04739046(Ⅱ)
		Glioma	yCD/mutTKSR39rep-ADP	NCT05686798(Ⅰ)
		Epithelial tumor	Anti-CD40	NCT05165433(Ⅰ)
	Oncolytic virus	Diffuse intrinsic pontine glioma	Non-secretory IL-12	NCT05717699(Ⅰ)
				NCT05717712(Ⅰ)
		Non-small cell lung cancer	MAGE-A3 and NY-ESO-1	NCT04908111(Ⅰ/Ⅱ)
		Solid tumor	OX40L	NCT04714983(Ⅰ)
		Epithelial tumor	Anti-CD40	NCT05165433(Ⅰ)
	Heart	Atrial fibrillation	KCNH2-G628S	NCT05223725(Ⅰ)
	Immune cells	HIV	The Cas9 system targeting HIV	NCT05144386(Ⅰ)
		SARS-CoV2	SARS-CoV-2 protein	NCT05526183(Ⅰ)
				NCT05094609(Ⅰ)
				NCT05373030(Ⅳ)
				NCT05515042(Ⅱ)
Lipid nanoparticles	Neuron	OTC deficiency	*OTC*	NCT05526066(Ⅱ)
	Immune cells	HIV	Native-like HIV-1 envelope trimer	NCT05903339(Ⅰ)
		SARS-CoV2	mRNA of the spike (S) protein	NCT04844268(Ⅰ)
		SARS-CoV2	*BNA1*59 mRNA	NCT05231369(Ⅰ)
		Influenza	H1ssF mRNA	NCT05755620(Ⅰ)
		Influenza	H1 HA mRNA	NCT05945485(Ⅰ)
	Lung	Cystic fibrosis	*CFTR* mRNA	NCT05712538(Ⅰ)
	Tumor	Non-small cell lung cancer	*TUSC2* gene	NCT05062980(Ⅰ/Ⅱ)
				NCT04486833(Ⅰ/Ⅱ)
		Solid tumor	mRNAs of OX40L, IL-23, and IL-36γ	NCT03739931(Ⅰ)

Note: OTC, ornithine transcarbamylase; *CFTR*, cystic fibrosis transmembrane conductance regulator; OTOF, otoferlin; HIV, human immunodeficiency virus; HSV, herpes simplex virus; TK, thymidine kinase; SARS-CoV-2, severe acute respiratory syndrome coronavirus 2; RPGR, retinitis pigmentosa GTPase regulator; hPDE6B, human phosphodiesterase 6B; VEGF, vascular endothelial growth factor; ATP7B, ATPase copper transporting beta; GDNF, glial cell-derived neurotrophic factor; AP4M1, adaptor-related protein complex 4 subunit mu 1; CYP21A2, cytochrome P450 family 21 subfamily A member 2; IL-2, interleukin-2; TNF-α, tumor necrosis factor-alpha; TUSC2, tumor suppressor candidate 2.

## DNA nucleases

### ZFNs and TALENs

**Figure 2:**
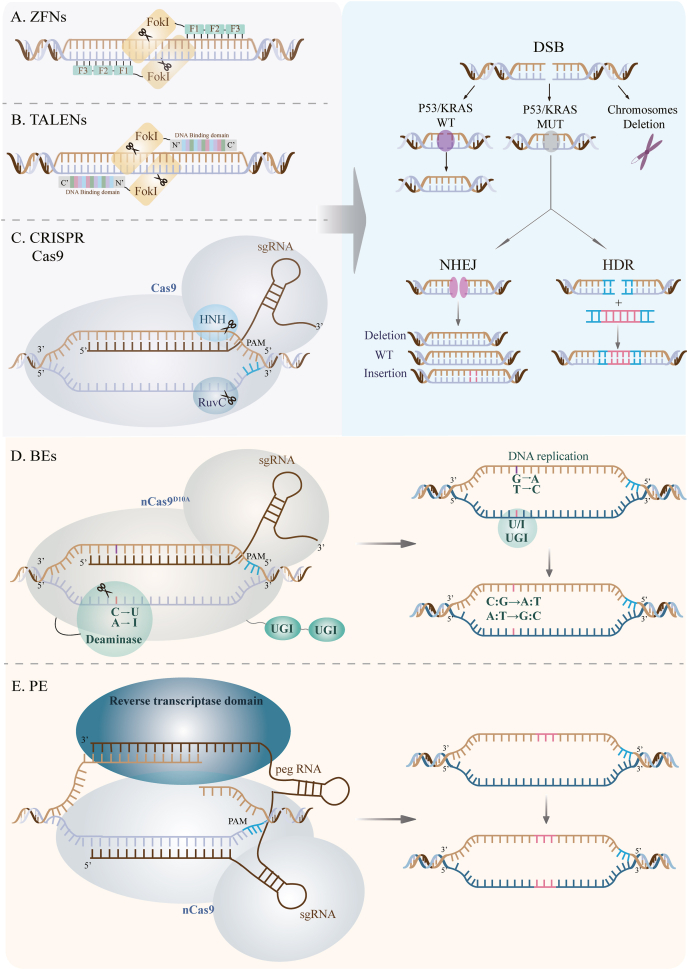
The summary of DNA nucleases. **(A, B)** Zinc finger nucleases (ZFNs) and transcription activator-like effect nucleases (TALENs) edit genome by programmable protein binding to targeting site. **(C)** Clustered regularly interspaced short palindromic repeats associated protein 9 (CRISPR-Cas9) edit genome by programmable RNA:DNA interaction. **(D)** ZFNs, TALENs, and CRISPR-Cas9 create double-strand DNA breaks (DSBs) initiating DNA-damage repair and defense response mediating by functional TP53 and KRAS, and increasing the risk of chromosome loss or truncation. **(E, F)** Base editors (BEs) and prime editors (PEs) edit genome DSBs cutting one strand of DNA, without creating DSB.

Zinc finger nucleases (ZFNs) (Fig. 2A) and transcription activator-like effector nucleases (TALENs) (Fig. 2B) were the first- and second-generation nucleases in DNA editing.^29^ The two nucleases editing the genome were based on programmable protein binding to specific DNA locations, producing DSBs with DNA endonuclease activity. This was followed by DSB repair through NHEJ or HDR to insert transgenes of interest.^11^ ZFNs and TALENs can edit genes *in vivo* for monogenetic mutation diseases, including hemophilia, Fabry disease, and epidermolysis bullosa.^30^, ^31^, ^32^, ^33^ Mitochondrial DNA (mtDNA) mutations can cause a series of diseases. However, it is challenging to deliver RNA inside mitochondria. Recently, Mok et al developed a CRISPR-free tool for mtDNA derived from an interbacterial toxin, DddA. The fusion of split-DddA halves, TALEN array proteins, and an uracil glycosylase inhibitor named DdCBE could localize at the target site and catalyze C:G to T:A conversions without an gRNA. DdCBE enables precise mtDNA manipulation while providing a tool for mtDNA mutation-related diseases.^34^ Further, Hu et al developed a TALEN-based BE tool for nuclear, mitochondrial, and chloroplast genome manipulation. This system is called cytidine deaminase–exonuclease–nickase–TALEN (CyDENT), a fusion of TALENs, a FokI nickase, a single-strand-specific cytidine deaminase, an exonuclease, and an uracil glycosylase inhibitor. The TALEN proteins guide the FokI nickase to a specific target site. The exonuclease excises a short patch of DNA, serving as a substrate for single-strand-specific cytidine deaminase and base editing. The editing efficiency of this system was 14%, with a strand specificity of 95%.^35^ However, designing and constructing a protein targeting a specific DNA site could be complex. During NHEJ repair, ZFN and TALEN gene editing showed a moderate rate of off-target insertions and insertions of other genes. Therefore, the safety profile of ZFNs and TALENs could be a primary concern for *in vivo* editing.^30^^,^^32^ Compared with ZFNs and TALENs, the CRISPR-Cas system simplified gene editing with lower off-target toxicities.^36^ As the third generation of nucleases, the CRISPR-Cas system accelerated the development of genetic engineering. This review details the CRISPR-Cas system-based gene editing.

### CRISPR-Cas system

In 2012, Jinek et al demonstrated that CRISPR-Cas9 could introduce DSBs at a specific DNA site using a dual-RNA-guided way (Fig. 2C).^37^ The CRISPR-Cas9 system possesses a Cas9 endonuclease, a mature CRISPR RNA (crRNA), and a trans-activating crRNA (tracrRNA). The crRNA has a 22-nucleotide segment near the 3′ end that is base-paired with tracrRNA, and the one at the 5′ end is a segment varying in sequence complementary to the target DNA. The tracrRNA triggers pre-crRNA processing and activates Cas9 to cut the crRNA-guided DNA strand.^36^ In the study, researchers designed a chimeric single RNA with a DNA recognition sequence and a hairpin structure generated by crRNA and tracrRNA base-paired sequence. With the single-guide RNA (sgRNA), Cas9 could target any DNA-specific site of interest by changing the recognition sequence.^36^^,^^37^ In 2013, Ran et al used the CRISPR-Cas9 system to edit eukaryotic cell genomes through NHEJ or HDR mechanisms to mediate gene knockouts or introduce genes of interest at specific sites.^36^^,^^38^ In 2013, Liu and Doudna demonstrated the off-target toxicity of RNA-programmable Cas9 nuclease through high-throughput sequencing. They suggested that shorter and less-active sgRNA with a low concentration of gRNA: Cas9 could enhance Cas9 nuclease specificity.^39^ Then, the CRISPR-Cas9 system was used in mammals to synthesize mice with genes of interest by editing fertilized eggs.^40^

CRISPR-Cas9 has helped explore gene functions and perform genome-scale screening of DNA and RNA with pre-made sgRNA libraries.^41^, ^42^, ^43^ In the coronavirus disease 2019 (COVID-19) pandemic, CRISPR-Cas9 helped probe the cellular factors related to severe acute respiratory syndrome coronavirus 2 (SARS-CoV-2). Various signaling pathways and proteins linked with the SARS-CoV-2 life cycle were revealed, delivering critical information for developing therapeutic strategies.^44^^,^^45^ CRISPR-Cas9 has also been employed to discover the interaction of different combinations of tumor suppressor gene mutations and identify the role of these gene combinations in tumorigenesis.^46^ Zmajkovic et al validated the pathogenesis of familial erythrocytosis, a gain-of-function mutation in erythropoietin (EPO) with CRISPR-Cas9.^47^ Similarly, CRISPR-Cas9 was massively used to parallelly trace cell lineage differentiation *in vivo* by making specific genetic scars inside original cells while detecting these scars at different stages or in mature cells.^48^

CRISPR-Cas9 has been applied to treat blood system monogenetic mutation disorders *ex vivo*. Transfusion-dependent β-thalassemia and sickle cell disease are disorders induced by a hemoglobin subunit beta (HBB) mutation. In 2015, Liang et al edited a mutant HBB gene, which led to β-thalassemia, using CRISPR-Cas9 and performed HDR with hemoglobin subunit delta (*HBD*) as a donor template within human tripronuclear zygotes.^49^ This research led to the clinical application of CRISPR-Cas9, although with a low HDR efficiency. The *BCL11A* gene represses γ-globin gene expression and fetal hemoglobin formation. Thus, *BCL11A* disruption can elevate hemoglobin subunit gamma (*HBG*) expression and compensate for *HBB* to produce fetal hemoglobin.^50^ In 2021, two patients with transfusion-dependent β-thalassemia and sickle cell disease were cured with autologous stem cell transplantation. CRISPR-Cas9 edited the HSPCs in these patients to disrupt the BCL11A gene enhancer.^27^ In another study, two patients with transfusion-dependent β-thalassemia achieved transfusion independence for at least 18 months after treatment of *BCL11A* enhancer-disrupted autologous hematopoietic and stem cell transplantation.^51^ In a phase 1/2 study, CRISPR-Cas9 helped disrupt the promoter of hemoglobin subunit gamma 1 (*HBG1*) and *HBG2* in HSPCs to promote fetal hemoglobin synthesis and produce normal red blood cells. Three patients were enrolled in the study, showing increased hemoglobin levels.^52^

Besides monogenetic mutation diseases, CRISPR-Cas9 was also applied to anti-tumor immunotherapy *ex vivo* and *in vivo*.^53^ Chimeric antigen receptor-T cell (CAR-T) immunotherapy is an effective tool for hematological malignancies and solid tumors, and CRISPR-Cas9 was widely used in CAR-T cell genome editing *ex vivo*. A phase I clinical trial of programmed death 1 (PD-1)-edited T cells with CRISPR-Cas9 for non-small-cell lung cancer achieved median progression-free survival of 7.7 weeks and median overall survival of 42.6 weeks.^54^ CRISPR-Cas9 helped edit allogeneic CAR-T cells by knocking out TCRα subunit constant chain (*TRAC*),^55^^,^^56^ beta-2 microglobulin (*B2M*), and *PD-1* genes of donor T cells to avoid graft-versus-host disease while mitigating host immune cell elimination of allogeneic CAR-T cells.^55^ In a phase I clinical trial, *ex vivo* CRISPR-Cas9 modified universal CD19/CD22 dual-specific CAR-T cells with TRAC and CD52 gene disruption. Thus, six patients with relapsed/refractory acute lymphoblastic leukemia achieved complete remission (83 %) without severe toxicities.^57^

However, the CRISPR-Cas9 system has drawbacks as it edits the genome by creating DSBs at target DNA sites. Moreover, DNA disruption would initiate a cellular repair and defense response. Ihry et al revealed that CRISPR-Cas9 induced toxicity and killed most HPSCs when DNA insertion or deletion efficiency reached 80% or higher. They predicted that the P53 pathway was involved in the CRISPR-Cas9 toxicity response.^58^ Haapaniemi et al have reported that cells with a functional P53 pathway correlate with lower genome editing efficiency. The HDR efficiency increased by inhibiting the P53 pathway to prevent the DSB damage response. These studies identified the role the P53 pathway played in CRISPR-Cas9 genome editing. Besides, KRAS hampers CRISPR-Cas9 genome editing by inhibiting Cas9-edited cells' growth.^59^ Thus, CRISPR-Cas9 would overcome the P53 pathway response by choosing cells with pre-existing *P53*-inactivating mutations and *KRAS* mutations. This would lead to accumulating *P53*-inactivated and *KRAS*-mutated cells and the risk of long-term irreversible results.^60^^,^^61^ Another safety concern of CRISPR-Cas9 was chromosomal loss and instability. Tsuchida et al established that Cas9-associated partial or entire target chromosome loss was a universal phenomenon, and T cells with chromosome loss could persist for weeks.^62^^,^^63^ Cullot et al observed that Cas9 induced large-scale chromosome truncations.^64^ Although chromosome loss could be mitigated to a certain extent by modifying the manufacturing process, these long-term genome toxicities should be monitored when Cas9-edited cells are applied to humans in clinical practice. The potential of CRISPR-Cas9-related genome toxicities promotes the need for and development of new DNA editing agents.

### BEs

Komor et al developed BEs (Fig. 2D) to precisely target one DNA base without introducing DSBs, including cytidine BEs (CBEs) and adenine BEs (ABEs).^65^ CBEs contain a fusion of Cas9 and cytidine deaminase enzyme, enabling the programming of the fusing protein by a guided RNA and mediating the target cytidine deamination, converting cytidine to uridine.^65^ Uridine has the property of base-pairing with thymine and converting C:G to A:T base pairs with a mismatch repair (MMR) mechanism in cells. The second and third generations of BE (BE2 and BE3) have uracil glycosylase inhibitor fusion and manipulate Cas9 to nick the non-edited strand (nCas9) to augment the base editing efficiency.^65^ To improve BE3 efficiency, a second uracil glycosylase inhibitor copy was added to BE3, forming BE4, and a bacteriophage Mu protein Gam was fused to BE4 (BE4-Gam) to decrease the indel formation risk to <1.5%.^66^ ABEs contain a fusion of nCas9 and adenine deaminase enzymes that convert A:T to C:G base pairs by deamination of adenine to inosine, and inosine is treated as guanine by polymerase. The base editing efficiency of the seventh generation of ABEs was 50%.^67^ Based on BE4 and ABE7.10, Koblan et al modified the nuclear localization signals, codon usage, and deaminase construction to enhance editing efficiency and formed BE4max and ABEmax.^68^ Several Cas9 variants were developed with different protospacer-adjacent motifs to target variable DNA sites and reduce undesired byproducts.^69^, ^70^, ^71^

BEs in the pool were used for high-throughput screening of single-nucleotide variants of genetic underpinnings of diseases, sensitivities to drugs, cellular physiology, and loss- or gain-of-function mutations.^72^, ^73^, ^74^ Besides screening, CBEs and ABEs have been used in preclinical research to treat single-nucleotide mutation disorders, including Hutchinson–Gilford progeria syndrome,^75^ spinal muscular atrophy,^76^ autosomal dominant hearing loss,^77^ sickle cell disease,^78^ and CD3δ severe combined immunodeficiency, *in vivo* or *ex vivo*.^79^ Grosch et al combined AAV9 and ABEs to correct mutations of the RNA-binding motif-20 (Rbm20) gene, a splicing factor of cardiomyocytes, *in vivo*. The Rbm20 gene mutation is the leading cause of aggressive dilated cardiomyopathy. A previous study showed that ABEs corrected >70% of mutated cardiomyocytes. After three months of gene editing, the cardiac function of mice achieved a level comparable to that of wild-type mice.^80^

Wild-type BEs can correct point mutations without producing DBSs. However, CBEs and ABEs cannot convert all bases, *e.g.*, C:G-to-A:T, C:G-to-G:C, T:A-to-A:T, and T:A-to-G:C.^11^ Moreover, undesired bystander mutations were observed, especially when multiple target nucleotides existed within the editing window. Off-target editing is also a major challenge we must overcome while base editing.

### PEs

PEs (Fig. 2E) were genome editing tools that could realize conversion or transition of all types of bases and insertion or deletion at specific target sites and were first reported in 2019 by Anzalone et al.^81^ PEs contain a nickase Cas9 fused with reverse transcriptase (RT), and a prime editing guide RNA (pegRNA). The pegRNA includes a spacer sequence to guide the PEs to the target site and a template encoding the sequence of interest. Cas9 nicks one of the DNA strands, and RT uses the pegRNA as a template to insert the sequence of interest at the target site. The first generation of PE (PE1) utilized the wild-type RT derived from Moloney murine leukemia virus (MMLV). It could introduce single-base substitutions, small insertions or deletions, with an efficiency of 0.7%–5.5%. PE2 introduced five mutations to wild-type MMLV RT and enhanced editing efficiency by 1.6- to 5.1-fold. PE3 used an additional sgRNA to direct PE3 to nick the non-edited strand and enhanced the editing efficiency to 55%.^81^ Chen et al and Hussmann et al revealed that DNA MMR inhibited prime editing and produced undesired indel byproducts. It was reported that interfering with MMR gene expression, particularly *the MLH1* gene, could improve prime editing efficiency. Hence, they designated PE2 or PE3 with *MHL1* truncation to develop PE4 or PE5, improving the efficiency by 7.7-fold and 2.0-fold compared with PE2 and PE3, respectively, and enhancing the product purity.^82^

Further, Lin et al reported that, besides PEs, pegRNA could also affect the editing efficiency. The optimum melting temperature of pegRNA is 30 °C, which could enhance the editing efficiency by 2.9-fold compared with PE2. They designed prime binding sites and used two pegRNAs in trans encoding the same edits for each DNA strand simultaneously, enhancing the editing efficiency by 17.4-fold.^83^ Choi et al designed a PE system with two pegRNAs, called PRIME-del, for long sequence deletion up to 10 kb with high efficiency and precision.^84^ Jiang et al utilized a PE system with paired pegRNAs to nick double DNA strands at target sites and insert sequences through RT templating the pegRNA. In this system, PE-Cas9-based deletion and repair resolve the replacement of gene length up to ∼10 kb and insertion of a desired sequence (up to 60 bp) without needing an exogenous DNA template.^85^ Anzalone et al have also designed a dual pegRNA system. They combined Bxb1 integrase into their research and realized gene-sized DNA plasmid (>5000 bp) integration to the genome and inversions of 40 kb targeted sequences in human cells without compromising the editing efficiency.^86^ In 2023, Doman et al developed a series of PE6 (a-g) variants utilizing phage-assisted continuous evolution. PE6a-g has a smaller size and 22-fold efficiency compared with PEmax. This leads to long sequence insertions into the brain and immune cells with 40% efficiency.^87^ Sun et al designed a PrimeRoot editor with enhanced PE and superior recombinases, leading to the precise insertion of large DNA sequences of up to 11.1 kb.^88^ Unlike BEs, which can only edit single bases for single-nucleotide variants, PE can correct 89% of genetically associated human diseases.^81^

PEs have been widely used in plant and animal genome editing.^89^^,^^90^ *In vivo* genome editing for genetically associated human diseases has been explored in hematopoietic stem cells, the brain, liver, heart, eyes, and tumors in animal models.^91^, ^92^, ^93^, ^94^ Li et al observed that transient inhibition of p53 could improve PE and CBE efficiency.^95^ However, PEs and BEs introduce genotoxic byproducts of deletion and translocation, though the frequency is lower than that of Cas9.^96^ PE is still in its infancy, and additional improvements should be made in editing efficiency, cargo loading capability, delivery strategies, and toxicity level for therapeutic applications.

### Delivery vehicles

**Figure 3:**
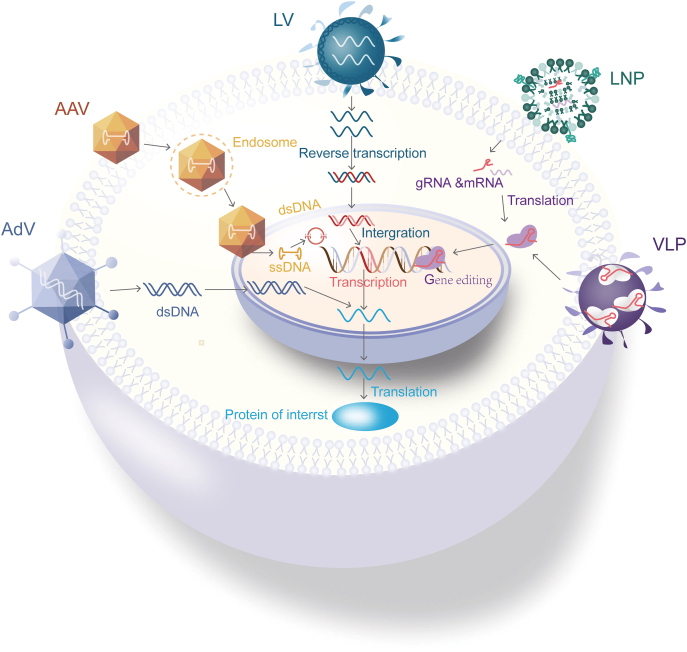
Overview of delivery vehicles. AdV, adenoviral vector; AAV, adeno-associated viral vector; LV, lentiviral vector; LNP, lipid nanoparticles; VLP, virus-like particle.

*In vivo* delivery includes viral vehicles, such as AAVs, adenovirus (AdVs), and lentivirus, and non-viral vehicles, such as LNPs and VLPs (Fig. 3).

### AAVs

AAVs were first separated from adenovirus preparation in the 1960s and became gene delivery vectors in 1984.^97^ The AAV genome is a ∼4.7 kb single-stranded DNA containing 60 copies of three viral protein (VP) subunits, VP1, VP2, and VP3. Two inverted terminal repeats (ITRs) flank both sides of the genome, encoding the rep, cap, and aap protein while signaling for replication and packaging.^98^ The AAV genome can integrate into the specific human genome at the AAV integration site 1 (AAVS1) on chromosome 19q13-qter.^99^, ^100^, ^101^ The capsid protein interacts with the receptor in the cell membrane, mediating AAV internalization. The receptors, called AAV receptors, are a series of N-linked glycosylated proteins.^102^ AAVs are identified with different serotypes based on the interactions between AAVs and AAV receptors, such as AAV1∼13.^102^, ^103^, ^104^ AAV2 is the most widely used serotype in clinical trials for its liver specificity,^105^ and AAV9 has the potency to infiltrate the blood–brain barrier and central nervous system gene delivery.^106^ Besides serotype, previous studies have explored the selective promoters of the liver,^107^, ^108^, ^109^ cardiomyocytes,^110^ muscles,^111^ neurons,^112^^,^^113^ *etc*., to improve AAV vector tissue specificity. The AAV genome is devoid of all these to maximize the carrying capacity and minimize immunogenicity and toxicity, except for ITRs. The recombinant AAVs (rAAVs) can carry ∼5.2 kb genes of interest.^114^

The first human trial of AAV was initiated in 1995 among adult patients with cystic fibrosis, an inherited autosomal recessive disease, and mild lung disease.^115^ The AAVs delivered the cystic fibrosis transmembrane regulator (CFTR) gene *in vivo*, which achieved a functional effect, with transgene persistence of up to 10 weeks *in vivo*.^116^ In 2000, the AAVs encoding blood coagulation factor IX (FIX) helped treat three adult patients with severe hemophilia B. The vector gene was observed in the muscles. The FIX level increased with a decreasing frequency of FIX infusion in patients.^117^ A clinical trial with rAAV2-FIX delivered through the hepatic artery was conducted. However, the persistence of FIX expression was short because of immunogenicity to the AAV capsid, causing the death of rAAV2-transduced hepatocytes.^117^ In subsequent clinical trials, rAAV therapy became pivotal in treating hemophilia.^109^^,^^118^, ^119^, ^120^, ^121^ In 2007, Kaplitt et al reported a phase I dose escalation trial of rAAV-GAD therapy for Parkinson’s disease. Here, 12 patients received unilateral subthalamic rAAV-GAD vector injections, and 10 of 12 patients demonstrated improvement in the Unified Parkinson’s Disease Rating Scale (UPDRS) score after three months of the injection; the improvement persisted for 12 months.^122^ Subsequently, Maguire et al reported rAAVs for Leber’s congenital amaurosis, an inherited blinding disease caused by retinal pigment epithelium 65 (RPE65) gene mutation. The rAAV vector carrying RPE65 cDNA was delivered in the subretinal membrane for 12 patients with Leber’s congenital amaurosis with a dose-escalation design. All the patients showed sustained improvement in objective and subjective vision measurements, though some side effects could be observed.^123^^,^^124^ In another clinical trial with rAAV-EPR65 for Leber’s congenital amaurosis, 6 of 12 patients showed improvements in retinal sensitivity for up to three years, declining from the peak at 6–12 months. Intraocular inflammation was observed in three patients, and clinically significant deterioration of visual acuity was observed in two patients.^125^^,^^126^ AAV gene therapy depicted impressive effects in genomic disorders, and the first AAV-based gene therapy with delandistrogene moxeparvovec (delandistrogene moxeparvovec-rokl; ELEVIDYS) was US FDA-approved in 2023.

AAVs are the most widely used vectors for *in vivo* gene therapy. However, the toxicity and limitations of AAVs are significant concerns. Recently, the death of a Duchenne muscular dystrophy patient six days after high-dose rAAV9 administration was reported. The leading cause of death was acute respiratory distress syndrome and severe diffuse alveolar damage.^127^ Transaminase elevation and liver damage were observed in liver-targeted AAV gene therapy.^117^^,^^126^, ^127^, ^128^, ^129^, ^130^, ^131^, ^132^

Furthermore, AAV-induced immune responses were also observed in retinal-targeted therapy.^126^^,^^133^ Besides, AAVs have a limited cargo-carrying capacity, and dual AAV delivery lowers efficiency and increases immunogenicity, accelerating the clearance of AAV vectors.^114^ Lastly, natural AAV antibodies shorten the lifespan of AAV gene therapy.^134^

### AdVs

AdVs were first discovered in the 1950s and observed in human adenoid tissue. AdVs were developed as a gene delivery vehicle in the 1980s.^135^ More than 120 species-specific AdV serotypes have been identified in nature.^136^ AdVs were non-enveloped viruses sized 65–90 nm with a double-stranded DNA genome flanked by two ITRs on both sides as packing signals. The genome contains nine fragments encoding late gene products (L1-5), transcription and translation gene products (E1-2, E4), and immune response regulation products (E3).^137^, ^138^, ^139^ When utilized as a gene vehicle, AdVs can remove all genomes while retaining the ITR structures. Thus, AdVs can carry ∼36 kb cargo without a strong immune response because of the absence of viral protein expression.^140^ AdVs are the most widely used vectors in gene therapy in clinical trials, possessing several advantages: i) large cargo packaging capacity; ii) easy manipulation and editing; iii) products of high titers on a large scale; and iv) high transduction rate.^141^

AdVs were primarily used as vectors of vaccines for infections or tumors. AdVs carried cDNA to somatic cells for transient expression of exogenous proteins, as they do not integrate into the genomes of most cell types.^25^^,^^142^, ^143^, ^144^ However, CRISPR-Cas9-based gene editing provides long-term gene correction with AdV vectors. In 2014, Ding et al utilized AdVs to express CRISPR-Cas9 and gRNA targeting proprotein convertase subtilisin/kexin type 9 (*PCSK9*) to induce a loss-of-function mutation within this gene *in vivo*. The results indicated that the gene editing efficiency was >50%, reducing serum PCSK9 and serum cholesterol by approximately 35%–40%.^145^ In 2017, they used the AdV vectors to carry CBE but not CRISPR-Cas9 to edit Pcsk9 *in vivo*, achieving >50% reduction in serum PCSK9 levels and a 30% decline in serum cholesterol.^146^ In 2018, Li et al designed a helper-dependent human CD46-targeting AdV carrying CRISPR-Cas9 to disrupt a repressor binding sequence within the γ-globin promoter. This led to a long-term transformation of β-to γ-globin expression.^147^ This was the first time of CRISPR-Cas9 *in vivo* editing of HSPCs. After three years, Li et al used an AdV vector to carry ABE or CBE targeting erythroid BCL11A or HBG1/2 promoter in HSPCs for monogenetic mutation diseases using an *in vivo* mouse model. The diseases included sickle cell disease and β-thalassemia. The results indicated that the gene editing efficiency was approximately 20%–40%, without severe toxicities.^148^ In 2023, Li et al employed PE with AdVs *in vivo* for editing hematopoietic stem cells to correct the *HBB* gene mutation for treating sickle cell disease. An average of 43% of mutated genes were corrected by *in vivo* PE5max editing (it was above the required curative threshold).^93^ The success of gene editing with CRISPR-Cas9 with CBE and PE in mouse models suggested the great potential of AdVs as an *in vivo* gene editing vector. However, the immunogenicity against AdVs is a significant challenge for its application in *in vivo* gene editing.^149^

### LVs

LVs were obtained from human immunodeficiency virus type 1 (HIV-1), modified as a gene vector in 1996.^150^ Subsequently, modifications were made to the standard platform of LVs, including three parental genes of gag, pol, and rev. These changes included adding a heterologous env gene and separating these genes with three plasmids to minimize the risk of vector self-replication.^151^ LVs can pack <10 kb of DNA cargo, sufficient to hold gene editing agents and donor templates. Besides, LVs can transfect dividing and non-dividing cells, thereby delivering nucleic acids to most somatic cells.^150^^,^^152^ Lastly, the tropism of LVs can be modified by altering the enveloping glycoprotein to target different tissues and cells.^153^^,^^154^ LVs helped edit HSPCs *ex vivo* to treat genetic diseases like severe combined immunodeficiency.^155^ Besides, LVs became valuable as vaccines for infectious diseases.^156^^,^^157^

LVs were used for *in vivo* gene delivery inside the liver and muscle cells in the 1990s.^158^ In 2021, Huckaby et al used LVs to produce CAR-T cells *in vivo*. They designed an LV-carrying CAR gene with a mutant Sindbis envelope, and a bispecific antibody redirected LV to CD3^+^ T cells. The *in vivo* production of CAR-T cells demonstrated robust anti-tumor efficacy in a mouse model.^153^ Compared with CAR-T cell production *ex vivo*, transfecting T cells with LVs targeting CD3^+^ T cells *in vivo* saves time and expense while expanding patient access to CAR-T therapy.^159^ Nicolas et al used LVs as vectors to carry human fumarylacetoacetate hydrolase (*Fah*) transgene into a hereditary tyrosinemia type-1 (HT1) mouse model. The results indicate that long-term genomic integration prevents HT1 complications with an efficacy comparable to standard therapy.^160^ Kerzel et al developed an LV vector with a macrophage-specific promoter for targeting macrophages in a live cancer microenvironment to deliver type I interferon, revealing anti-tumor activity against liver metastases.^161^ Lee et al developed E1E2-pseudotyped LVs carrying Cas9 and sgRNA targeting kinesin spindle protein (KSP) to tumor cells in mice. The LVs, redirected to Huh7 cells due to E1E2 binding to the receptor on the cell membrane, disrupted the *Ksp* gene and inhibited tumor growth.^154^ Suh et al applied LVs to deliver ABE and a sgRNA targeting the Rpe65 gene mutation for Leber’s congenital amaurosis, with a gene correction efficiency of 29% and improved retinal and visual function.^162^ LVs were widely applied *in vivo* for genomic integration; however, there are few reports of their use for gene editing *in vivo*.

### LNPs

LNPs were developed in the 1990s as an emergency in nanotechnology. Liposomes, developed in the 1965s, were considered the first generation of LNPs because of their nanosized structures.^163^ LNPs use nanofabrication to synthesize the particles in nanoscale structures (0.1–100 nm). They behave as carriers of DNA nucleases with desired properties and performance, becoming reliable and reproductive in large quantities at low cost.^164^ LNPs are in the spotlight because of the COVID-19 mRNA vaccine. LNPs are vital in mRNA protection and transportation.^165^ Liposomes were first used as a drug delivery vehicle for small drugs, such as anti-cancer drugs with low solubility. Doxil, liposomal doxorubicin, is the first liposome-modified product approved by the US FDA in 1995, lengthening the half-life of doxorubicin and decreasing its cardiotoxicity.^166^ Subsequently, LNPs were widely used in anti-cancer, anti-fungal, analgesic, mRNA vaccine, viral vaccine, immunosuppressant, antihemophilic, respiratory, and hormonal drugs. The most used nonviral delivery system for carrying nucleic acids is the cationic LNPs, which include ionizable cationic lipids, cholesterol as stabilizers, helper lipids, and polyethylene glycol (PEG)-lipid.^167^ Ionizable cationic lipids are uncharged in the bloodstream and positively charged in the endosomal tissue with a pH of 5.0 and have lower toxicity than nonionizable lipids.^168^ To enhance the physical stability, loading capacities, and bioavailability of LNPs, the next generation of solid LNPs (SLN), nanostructured lipid carriers (NLC), and cationic lipid-nucleic acid complexes were synthesized and modified.^165^^,^^169^, ^170^, ^171^ LNPs could deliver hydrophilic drugs in the aqueous interior of liposomes and hydrophobic drugs between the hydrocarbon chains of lipid bilayers. Here, the function of LNPs in nucleic acid delivery and gene therapy *in vivo* is primarily discussed.

Patisiran is the first approved LNP for delivering RNA interference (RNAi) drugs for hereditary transthyretin amyloidosis.^172^, ^173^, ^174^ In 2021, the US FDA approved two COVID-19 mRNA vaccines, BNT162b2 and mRNA-1273, by Pfizer/BioNTech and Moderna, respectively. These are the latest successes of LNPs.^175^ Besides mRNA vaccines, Palanki et al have attempted to deliver ABEs with LNPs to the perinatal brain for congenital brain disease in a mouse model, demonstrating a proof-of-principle for LNPs delivering gene editing tools to the brain.^176^ Sela et al developed SynO4 LNPs for Parkinson’s disease (PD), tagged with transferrin to target the transferrin receptors on the blood–brain barrier after PD overexpression. The results indicated that the transferrin-tagged SynO4 LNPs improved mouse motor function and learning ability without severe toxicities.^177^ Steinman et al reported that cyclopropenium nanoparticles are a carrier for plasmid DNA, showing high efficiency and minimal toxicities.^178^

There are four primary challenges that LNPs face with *in vivo* delivery. First, the efficiency of LNP delivery *in vivo* is still low. Serum and pH values in the endosomal membranes are the main factors affecting mRNA LNP efficacy. As researchers observed, when the endosomal membrane pH is lower than 6, the LNP binding to the endosomal membrane and LNP disintegration sharply increase. This was observed through surface-sensitive fluorescence microscopy with a single LNP resolution. LNPs preincubated in serum formed a protein corona that would impact LNP binding to the endosomal membranes. mRNA LNP intake increased as lipoprotein was depleted from the serum.^179^ Cui et al revealed that loading more mRNA per LNP could improve mRNA cytosolic delivery, the mechanism of which lies in that the particle with more mRNA has a larger size and a larger hydrophobic surface with more hemolytic activity to form a larger protein corona in the serum. This could lead to more accumulation inside macropinocytosomes.^180^ Dobrowolski et al revealed that lipidoids with one carbon branch improved efficacy ten-fold due to the strong surface ionization at a late endosomal pH of 5.0. Besides LNP structure and parameters, target cell heterology is a major factor impacting mRNA delivery efficacy.^181^ Radmand et al analyzed the transcriptional response with single-cell RNA sequencing. The results suggested that the efficacy of LNPs is affected by LNPs and the endogenous RNA of the target cells and proteins associated with RNA or protein metabolism. Therefore, it is essential to identify the cell types that can be ideal targets.^182^ Yu et al have designed LNPs with ionizable amino-lipids and inverse cubic and hexagonal mesophase transition, enhancing the efficacy of mRNA delivery into lung macrophages.^183^

Second, target delivery *in vivo* is challenging. There are countless ways for the nanoparticles to interact with the cells *in vivo*, leading to unexpected and hazardous toxicities.^184^ Jiang et al designed a nebulized mRNA LNP with stability during nebulization and penetrating ability through cellular or extracellular barriers to enhance targeted drug delivery. The formulation of nebulized LNPs offers an alternative LNP delivery, which has significant meaning in respiratory diseases.^185^ Fei et al designed mannosylated LNPs carrying miR-146a that could preferentially bind to alveolar macrophages *in vitro*. They used the mannosylated LNPs to treat acute respiratory distress syndrome induced by hemorrhagic shock, dramatically mitigating lung inflammation.^186^ Qiu et al demonstrated that compared with conventional LNPs with an ester bond at the O-terminal, LNPs with an amide bond at the N-terminal could target mouse lungs for mRNA delivery. Moreover, these LNPs could target different subtypes of pulmonary cells by changing the headgroup structure. With these LNPs for tuberous sclerosis 2 (Tsc2) mRNA delivery to the lungs, researchers successfully treated lymphangioleiomyomatosis, a monogenetic disorder induced by Tsc2 gene loss-of-function mutations.^187^ Shen designed a spleen-targeted LNP that used a decationizable quaternium lipid-like molecule (lipidoid), possessing a positive charge in physiological conditions, enhancing the storage stability. On encountering esterase in the serum, the LNP promptly acquires a negative charge to deliver mRNA to the spleen, which mediates the ovalbumin (OVA) mRNA specifically expressed in spleen cells.^188^ LNPs delivered CAR mRNA to myeloid cells and reprogrammed myeloid cells to CAR-macrophages that showed anti-tumor efficacy, indicating the potential of LNP immunotherapy against tumors *in vivo*.^189^^,^^190^ Sun et al designed an LNP-miR130b antagomir to treat diffuse large B-cell lymphoma by down-regulating miR130b, inhibiting Th17 recruiting, and promoting lymphoma cell autophagy.^190^

Third, the cargo size of LNPs is limited. Therapeutic RNAi and mRNA are the most common nucleic acid cargoes of LNPs.^191^^,^^192^ Yin et al employed LNPs to deliver Cas9 mRNA, AAV encoding sgRNA, and DNA templates to mice to rectify fumarylacetoacetate hydrolase (Fah)-splicing mutation while treating tyrosinemia and achieved 6% correction.^193^ Later, Song et al used LNPs to deliver ABE mRNA and a sgRNA targeting the liver while correcting A > G splice-site mutation of the fumarylacetoacetate hydrolase (*Fah*) gene in the liver, achieving high efficiency in adult mice.^194^ Further, Rothgangl et al employed LNPs to deliver an ABE and a gRNA targeting *Pcsk9*, a negative regulator of low-density lipoprotein receptor, to treat familial hypercholesterolemia induced by a gain-of-function mutation of PCSK9. The results indicated that the ABE LNPs induced 67% and 34% gene editing in mice and macaques, leading to 95% and 32% of plasma PCSK9 expression and 58% and 14% of low-density lipoprotein level decrease, respectively. This research shows that LNPs are an alternative platform for Cas9-based gene editing.^195^ Rodríguez-Gascón et al designed a galactomannan (GM)-tagged SLN to deliver the plasmid DNA of α-galactosidase A (α-Gal A) to the liver for the treatment of Fabry disease, which is a monogenetic disease. *In vitro* and *in vivo* results showed that with GM-SLN, α-Gal A activity was increased in the HepG2 cell line and in plasma, liver, heart, kidneys, and other organs of mice with Fabry disease.^169^ This work highlighted the potential of LNPs in pDNA-based gene modifications. However, the optimal lipid composition for RNA and pDNA varies, and pDNA delivery efficiency is impacted by lipid composition. Qin et al observed that lipid composition of 39:10:50:1 mol% for DLin-KC2-DMA:DOPE:cholesterol:C16-PEG2000 can provide high-efficiency delivery of siRNA, pDNA, or their combination.^196^

Fourth, the immunogenicity of LNPs is high *in vivo*. The LNPs should have a reduced immune response and unwanted absorption to achieve a long life span and stealth *in vivo*. PEG coating (PEGylation) is the most widely used approach to realize LNP stealth among US FDA-approved drugs.^197^ However, as PEG is nondegradable, it accumulates in the kidney and spleen, resulting in macrophage formation and epithelial vacuolization, and inducing anti-PEG antibodies. This could accelerate LNP clearance and cause allergic reactions and other chronic diseases.^198^, ^199^, ^200^ Sun et al developed a neutral α-helical polypeptide, poly(γ-(2-(2-(2-methoxyethoxy)ethoxy) ethyl (l)-glutamate), called (L)-P(EG(3)Glu), as a substituent of PEG. (L)-P(EG(3)Glu) showed lower immunogenicity than PEG and is potentially valuable for the clinical use of LNPs.^201^

### VLPs

VLPs refer to a series of protein particles that self-assemble to form capsids, cores, and envelopes, which have a virus structure without nucleic acid.^202^ Thus, VLPs possess no risk of infection-like viruses. VLPs have repetitive surfaces to stimulate B-cells as pathogen-associated structural patterns (PASPs). VLPs have been widely used to express virus proteins as vaccines, *e.g.*, HBV vaccine, HPV vaccine, malaria, COVID-19, and even tumors.^203^ VLPs are vehicles of gene editing, delivering nucleases like Cas9 RNP and ABE.^204^, ^205^, ^206^, ^207^, ^208^, ^209^ Lyu et al observed that “Gesicle”, a VLP containing CherryPicker red, VSV-G, and Cas9/sgRNA ribonucleoprotein (RNP) targeting HIV, could inactivate HIV in microglial cells.^205^ Mangeot et al applied lentiviral-like particles (LVLPs) for Cas9 RNP delivery. They achieved an on-target indel efficiency of 35.5%, with a lower off-target indel rate than mRNA LVLP delivery. Mangeot et al used LVLPs to deliver RNP in mouse embryos and liver while observing gene indel in mice.^206^ Further, Gee et al used VLPs to deliver Cas9 RNP *in vivo* to induce permanent exon skipping for Duchenne muscular dystrophy therapy. This indicated the potential of VLPs for gene therapy *in vivo*.^207^ Hamilton et al used VLPs in immunotherapy by delivering Cas9 RNP and CAR gene into T cells *ex vivo* to synthesize CAR-T cells.^210^ Yao et al used the RNA aptamer and aptamer-binding protein (ABP) structure to enrich Cas9 RNP in the VLPs by inserting the RNA aptamer into sgRNA and fusing ABP to both terminals of exosome transmembrane protein CD63.^211^ They applied this structure in VLPs to deliver ABE RNP, which showed high on-target base editing in human cells.^212^ Although VLPs have been employed in gene editing *ex vivo* and *in vivo*, they have modest efficiency with limited therapeutic efficacy. In 2022, Banskota et al developed an engineered VLP, which enhanced protein loading by 16-fold and base editing efficiency by 8-fold compared with previous VLPs. With this engineered VLP, they edited the PCSK9 gene *in vivo* with a 63% decrease in liver gene editing and a 78% reduction in serum Pcsk9 level. Besides, mice with genetic blindness were treated while partially restoring their visual function.^213^ The impressive results indicated the promising application of engineered VLPs as a gene therapy vehicle. Haldrup et al developed VLPs to deliver BEs and PEs via gene editing *in vivo*, enabling donor-free BE and PE gene editing.^214^

## Conclusions and future perspective

*In vivo* gene therapy can potentially cure genetic diseases. However, there are challenges before it can be approved for clinical use. First, efficiency and safety are significant concerns for *in vivo* gene therapy. With the development of DNA nucleases and delivery vectors, gene therapy *in vivo* has achieved high efficiency and low off-target toxicities. However, target delivery is limited among the current delivery vectors. Second, monogenetic mutation diseases that have been clinically or pre-clinically treated with *in vivo* gene therapy are just the tip of the iceberg. Thousands of monogenetic diseases requiring treatment remain, and *in vivo* gene therapy is their most promising strategy. Lastly, *in vivo* gene therapy can be expanded to cancers, auto-immune diseases, infections, and other degenerative diseases other than genetic mutation diseases. Thus, *in vivo* gene therapy will be a powerful weapon for treating a broad spectrum of diseases in the future.

## CRediT authorship contribution statement

**Tao Wang:** Writing – original draft, Conceptualization. **Mingyang Yu:** Writing – original draft. **Ping Liu:** Visualization, Writing – original draft. **Zhiqiang Song:** Writing – review & editing. **Cheng Li:** Writing – review & editing, Conceptualization. **Jianmin Yang:** Writing – review & editing, Conceptualization. **Na Liu:** Writing – review & editing, Conceptualization.

## Funding

This work was supported by the 10.13039/100012905Shanghai Science and Technology Development Funds (China) (No. 23YF1458800), 10.13039/501100002858China Postdoctoral Science Foundation (No. GZC20233559, 2023M744286), Research Fund for the Basic Medical Research for the Youth of the Changhai Hospital (2023QD24), and Research Fund for the Basic Medical Research for the Youth of Naval Medical University (2024QN024), Special Program for Clinical Medicine Research (2024LYC015), Research Fund for the Basic Medical Research for the Youth of the 10.13039/501100008866Changhai Hospital (Shanghai, China) (No. 20230024), and the 10.13039/501100001809National Natural Science Foundation of China (No. 82100162, 82270202).

## Conflict of interests

The authors have no competing interests to declare.
